# PRESIDENTIAL ADDRESS: THE ETHICS OF RECOGNITION, RESPONSIBILITY, AND RESPECT

**DOI:** 10.1111/j.1467-8519.2009.01763.x

**Published:** 2009-09-29

**Authors:** Matti Häyry

**Keywords:** ethics, bioethics, practice, study, recognition, responsibility, respect

## Abstract

Ethics can be understood as a code of behaviour or as the study of codes of behaviour. While the mission of the International Association of Bioethics is a scholarly examination of moral issues in health care and the biological sciences, many people in the field believe that it is also their task to create new and better codes of practice. Both ways of doing bioethics are sound, but it is important to be aware of the distinction. In this paper, I will study the sources and aims of ethics and suggest a code of conduct for bioethicists based on recognition, responsibility, and respect.

My message in this presentation is simple.^1^ We could listen to each other more carefully; we could be more understanding of each other's motivations, needs, and values; and we could try to be more respectful of each other's moral views and methodological choices. It would, in fact, make a lot of sense if we did all these things. I will try to convey this message by answering four questions, namely: What is ethics? How is ethics practised? What do I mean by recognition, responsibility, and respect? And how can recognition, responsibility, and respect be made to count in bioethics?

## WHAT IS ETHICS?

The word ‘ethics’ means, in many languages, two distinct things: a *code of behaviour* and the *study of codes of behaviour*.

‘Codes of behaviour’ can include ethical theories like Kantianism and utilitarianism, but also ideologies, professional self-regulations, and traditions. The protestant work ethic described by Max Weber meant the way in which people operate in capitalist societies – by and large, statistically, or as ideal types. Medical ethics is the way in which health professionals conduct themselves, or feel that they ought to conduct themselves. The Croatian way of life is the way in which Croatian people behave, or feel that they ought to behave.^2^

The origin of these and other codes of behaviour can be found in various directions, including biology, tradition, religion, culture, economy, technology, politics, laws and regulations, art and entertainment, reason, relationships, and emotions. They are thought to bind, or oblige, people either objectively, subjectively, or intersubjectively. God, Nature, Reason, and Emotion have been seen as objective, external forces that morally require us to act in certain manners. Individuals' attitudes and desires, by contrast, have been regarded as subjective, internal powers that motivate and guide us in more personal ways. Naturally evolved habits and customs and voluntarily developed contracts are forms of collective, intersubjective ethical control. Objective obligations are often believed to be universally binding, whereas subjective and intersubjective forces have been seen as relative to persons, communities, or cultures.

Ethics in the other sense, the ‘study of codes of behaviour’, is the area in which the International Association of Bioethics is designed to operate. According to the Constitution of the Association, bioethics is the ‘study of ethical, social, legal, philosophical, and other related issues arising in health care and the biological sciences.’ The approaches used in this study can vary considerably, as we know from the experience of our biennial world congresses, comprising at least the sociological, psychological, biological, and genetic; historical, theological, philosophical, and aesthetic; and legal, gender-driven, economic, and ecological. Ethics as an academic discipline has, however, one, and only one, aim: *to acquire knowledge and gain understanding*. The knowledge acquired and understanding gained can be seen either as its own end or as an instrument to other ends, such as the facilitation of practical decision making, but bioethics in this sense is, by definition, not intended to produce rules or regulations.

## HOW IS ETHICS PRACTISED?

Depending on the meaning given to the word, ethics can be practised by doing or by studying. *Doing* ethics involves the creation, interpretation, and enforcement; or the revision, criticism, or rejection of codes of behaviour. Studying ethics involves the examination of these constructive and critical ways of doing ethics; and the analysis of the nature and implications of different behavioural codes. The areas in which codes are deliberately done and undone are politics, legislation, art, and entertainment; while biology, tradition, and other background factors can act either for or against conscious human attempts. The only way to *study* ethics is to use scholarly methodologies to gain data and comprehension: producing norms or imposing values forms no part of academic bioethics as such.^3^

The last few decades have seen the rise of a new semi-professional group: ‘bioethicists’. Some of these people (that is, some of us) practise ethics in the first sense, doing and undoing codes; others practise ethics in the second sense and study codes. Many are confused, perhaps wanting to do one but being pressed to do the other. This can work in both directions: academics can be required to construct frameworks for regulation; and medical and legal professionals can be expected to anchor their practices in knowledge and understanding of general ethics instead of the traditions of their trade. Most bioethicists simply do both, oblivious to the distinction.^4^

Confusion concerning what bioethicists ought to do has the potential for breeding impolite disagreement. People who study ethics from one viewpoint and believe that it is also their job to create substantive rules are prone to say, ‘If you don't believe in autonomy and efficiency, you are irrational.’ Others who disagree with them normatively but hold the same view of their role in regulation are likely to respond, ‘If you don't believe in dignity and solidarity, you are immoral.’ Still others, disagreeing with both ethical views presented, can observe, ‘If you believe in either, you are intellectually lame – better theory is needed.’ And a final group, distancing themselves from theoretical constructions, can comment, ‘If you believe in any of these, you are culturally insensitive – local traditions and inborn practices are paramount.’ Amidst all these views, we could use some recognition, responsibility, and respect.

## WHAT DO I MEAN BY RECOGNITION, RESPONSIBILITY, AND RESPECT?

Recognition, responsibility, and respect have many technical and theoretical meanings in philosophical and ethical debates. Competing outlooks on these notions often breed impolite disagreement, so I am evoking only the most rudimentary everyday connotations of the words. To recognize you, to be responsible for your, and to respect you, entails that I do not ignore your views, your needs, or your will.

*Recognition*, in some more detail, means noticing people, their views, and their actions; understanding their objectives and the ways in which they pursue them; and taking them into account in all ethical decisions. The ‘taking into account’ element of recognition can lead in two opposite directions.^5^ The first is resolving to bring about changes in people, their codes of behaviour, or their thinking – this ‘pulling element’ flowing from recognition is responsibility. The second is resolving to keep away from people, their codes, or their thinking; and to not bring about changes – this ‘pushing element’ flowing from recognition is respect.

*Responsibility* can arise from emotions of empathy (‘I could be in their position’) or guilt (‘It is my fault that they are in that state’); from economic thinking (‘Healthy citizens cost less and are more productive’); or from a sense of obligation (‘I am a health professional and they are in my care’). This attitude can translate into good sensitive actions aimed at promoting health, regulation, and thinking. It can also translate into paternalistic control over people's lives, codes, and ideas against their own values and wishes.

*Respect*, in its turn, can be motivated by concern for the liberty and values of persons (‘Their lives, their choices’); or it can be motivated by fear of opposition, blame, or retaliation (‘Better do nothing, lest they turn against me’). As in the case of responsibility, respectful behaviour can have two faces. It can be (or be seen as) good deferential abstinence from unnecessary interventions into people's lives and thoughts. It can also be (and be seen as) callous negligence in the name of individual freedom and self-determination.

The ethics of recognition, responsibility, and respect is a professionally normative view suggesting that in practising ethics, both as ‘doing’ and as ‘studying’, we would do well to abide by three standards.^6^ We should pay attention to and try to understand people and their actions, values, and norms. We should pay attention to and consider acting upon the pulling effect of people's needs, codes, and ideas. And we should pay attention to and consider refraining from action prompted by the pushing effect of people's values and ideals.

## HOW CAN RECOGNITION, RESPONSIBILITY, AND RESPECT BE MADE TO COUNT IN BIOETHICS?

Recognition, responsibility, and respect can be applied to both main forms of bioethics – to ‘doing’ codes by law, politics, committee work, consultation, art, entertainment, and ‘philosophy’*and* to studying bioethics by academic bioethics, international collaboration exemplified by what is best in our congresses, and open-ended committee work.^7^

It is probably good for legislators and other code-creators to notice people and understand what their goals and ways of pursuing them are; to assume a commitment to see to people's needs equitably and protect their ways of life; and to be mindful of people's integrity and of values like autonomy, dignity, happiness, and humanity. I cannot say these things categorically, though, because there are many other views on the purpose and proper way of giving laws and setting rules of behaviour.

What I can claim categorically is that it would be good for us academic bioethicists to notice our colleagues and understand what their goals and ways of pursuing them are; to assume a commitment to state our own views strongly and to criticize those of others fairly; and to be mindful of our own academic and personal integrity as well as the integrity of our rivals.

In conclusion, bioethics means both doing and studying codes of behaviour – and keeping these apart would be a start. The recognition of people's goals and ways, and responsibility and respect flowing from this, would probably help in the creation of sensible codes and it would certainly help in a polite quest for understanding them.

## Footnotes

1 The following account duplicates more or less word for word my presidential address as it was delivered at the International Association of Bioethics Ninth World Congress of Bioethics in Rijeka, Croatia on 8 September 2008. During the discussion period, many colleagues offered their comments, all of which I am grateful for and some of which I have included in the footnotes of this article, with my responses. I am also grateful to all those who helped me to improve this presentation by observations on its earlier versions, given at the Annual International David Thomasma Bioethics Retreat in Paris, France on 26 June 2008, and at the 22^nd^ European Conference on Philosophy of Medicine and Health Care in Tartu, Estonia on 21 August 2008.

2 Professor Paul Ulhas Macneill commented that the use of the term ‘code of behaviour’ can lead to the interpretation that ethics cannot be a device of personal self-development. This is not what I meant. Although not included in my examples, a person's own ethical integrity can for the purposes of this article be seen as that person's ‘code of behaviour’, to be cultivated and examined like the more collective codes.

3 Commenting primarily on the ‘doing’ part of bioethics, Professor Solomon Benatar observed that the degree to which codes of behaviour come to be imposed on people depends on whether power (to implement rules in this case) is used *with* people or *over* people. I agree and think that my ideas on recognition, responsibility, and respect could facilitate thinking about codes ‘with’ people.

4 Professor Jing-Bao Nie and Mr Mark Cutter made strong cases for the practicality of bioethics, arguing among other things that practical relevance is professionally required of bioethicists. Formative training can influence the way in which academics see this issue. Medical and legal qualifications can tip the balance in the professional and practical direction for my two commentators, while as a philosopher I tend to safeguard the separateness of the ‘knowledge’ side of ethics. Then again, taking a cue from Professor James Dwyer's remarks on pragmatism, perhaps the ultimate test for truth of a thing is that ‘it works’, in which case practice is needed for the detection of true knowledge. My main point, though, is that it is possible and helpful to make the distinction into practical and theoretical in bioethical activities.

5 This useful division was pointed out to me by Professor Rosamond Rhodes at the Annual International David Thomasma Bioethics Retreat in Paris, France on 26 June 2008.

6 The expression ’professionally normative view’ is not ideal in this context, because bioethics is not a genuine profession and because normative views are usually thought of as prescriptive to those who act in real-life situations rather than to those who create rules for them or study such rules. My original wording in the address was ‘metaethical view’, but, as noted by Professor Susan Dodds, this is also misleading since a prescription, albeit only to bioethicists, is clearly involved.

7 Since philosophy as an academic discipline belongs to the ‘study’ part of bioethics, ‘philosophy’ in scare quotes refers to the activities of scholars who believe that they should participate in legislation and regulation in roles other than that of an academic expert. By ‘open-ended committee work’ I mean public endeavours examining the background of issues for legislation and regulation without prescribing an absolute course of action.

